# Impaired effective functional connectivity in the social preference of children with autism spectrum disorder

**DOI:** 10.3389/fnins.2024.1391191

**Published:** 2024-05-30

**Authors:** Simin Deng, Si Tan, Cuihua Guo, Yanxiong Liu, Xiuhong Li

**Affiliations:** ^1^School of Public Health (Shenzhen), Shenzhen Campus of Sun Yat-sen University, Shenzhen, Guangdong, China; ^2^Department of Child Preventive Care, Dongguan Children’s Hospital, Dongguan, Guangdong, China; ^3^Department of Maternal and Child Health, School of Public Health, Sun Yat-sen University, Guangzhou, Guangdong, China

**Keywords:** autism, social preference, fALFF, GCA, children, MRI

## Abstract

**Background:**

The medial prefrontal cortex (mPFC), amygdala (Amyg), and nucleus accumbens (NAc) have been identified as critical players in the social preference of individuals with ASD. However, the specific pathophysiological mechanisms underlying this role requires further clarification. In the current study, we applied Granger Causality Analysis (GCA) to investigate the neural connectivity of these three brain regions of interest (ROIs) in patients with ASD, aiming to elucidate their associations with clinical features of the disorder.

**Methods:**

Resting-state functional magnetic resonance imaging (rs-fMRI) data were acquired from the ABIDE II database, which included 37 patients with ASD and 50 typically developing (TD) controls. The mPFC, Amyg, and NAc were defined as ROIs, and the differences in fractional amplitude of low-frequency fluctuations (fALFF) within the ROIs between the ASD and TD groups were computed. Subsequently, we employed GCA to investigate the bidirectional effective connectivity between the ROIs and the rest of the brain. Finally, we explored whether this effective connectivity was associated with the social responsiveness scale (SRS) scores of children with ASD.

**Results:**

The fALFF values in the ROIs were reduced in children with ASD when compared to the TD group. In terms of the efferent connectivity from the ROIs to the whole brain, the ASD group exhibited increased connectivity in the right cingulate gyrus and decreased connectivity in the right superior temporal gyrus. Regarding the afferent connectivity from the whole brain to the ROIs, the ASD group displayed increased connectivity in the right globus pallidus and decreased connectivity in the right cerebellar Crus 1 area and left cingulate gyrus. Additionally, we demonstrated a positive correlation between effective connectivity derived from GCA and SRS scores.

**Conclusion:**

Impairments in social preference ASD children is linked to impaired effective connectivity in brain regions associated with social cognition, emotional responses, social rewards, and social decision-making. This finding further reveals the potential neuropathological mechanisms underlying ASD.

## Introduction

Autism spectrum disorder (ASD) is a lifelong neurodevelopmental disorder with a prevalence of approximately 1 in 36, which is increasing every year (Maenner et al., 2023). It is characterized by social communication deficits, stereotyped or repetitive behaviors, and restricted interests. Among these features, social communication deficits are the most common features of ASD (Tager-Flusberg, 2010). These significantly impact an individual’s preference for social stimuli. Social preference refers to an individual’s ability to perceive, evaluate, and prioritize social stimuli. This ability plays a crucial role in complex social interactions and the establishment of meaningful social relationships (Fortier et al., 2022). Research has shown that individuals with ASD exhibit atypical social preference, which is associated with abnormal neural activation and network connectivity in brain regions related to social cognition, emotional responses, social rewards, and social decision-making processes (Zhou et al., 2019; Kelly et al., 2020). Notably, alterations in the medial prefrontal cortex (mPFC), amygdala (Amyg), and nucleus accumbens (NAc), brain regions known to be part of a neural circuit responsible for social preference, have been observed in individuals with ASD (Bault et al., 2011; Gangopadhyay et al., 2021). These findings provide crucial insight into the neurobiological mechanisms of ASD. We have termed the collection of brain regions involved in driving social behaviors and preferences in humans “brain regions associated with social preference.”

This collection involves complex interactions and coordination among multiple brain regions associated with social cognition, emotional responses, social rewards, and social decision-making. As mentioned before, three specific brain regions that play crucial roles in the network are (1) the mPFC that is involved in social cognition, emotional regulation, and social interaction rewards (Neubert et al., 2015), (2) the Amyg that contributes to social cognition, social rewards, and social decision-making (Putnam and Chang, 2021), and (3) the NAc that is essential for motivation and behavior coordination in reward acquisition. Numerous studies have indicated that the mPFC exhibits heightened activity during social bonding in rodent species (Lee et al., 2016), social preference behavior in non-human primates (Chang et al., 2013), and observations of social interactions among conspecific animals (Sliwa and Freiwald, 2017). On the contrary, studies on ASD patients have found that these individuals exhibit decreased activation in the mPFC and lower levels of social reward following positive social interactions (Sumiya et al., 2020). These findings suggest that the mPFC potentially reduces the motivation for social interaction by inhibiting social-reward brain activity in individuals with ASD, leading to a decrease in social preference. Furthermore, additional studies have observed that the reduced social preference in individuals with ASD is likely associated with decreased reward processing for social engagement in the NAc, along with increased reward processing for non-social pursuits, which together manifest as narrow interests. Decreased amygdala activation may lead to changes in the social reward process, resulting in decreased social preference in individuals with ASD (Damiano et al., 2015).

Although we have recognized the crucial roles of the mPFC, Amyg, and NAc in the atypical social preference observed in ASD, there is still limited knowledge of their effective functional connections. To gain a more comprehensive understanding of the pathophysiological process of social preference in ASD, it is imperative to delve into how mPFC, Amyg, and NAc drive the activity of other regions, and detect any alterations in the feedback mechanism when other parts of the brain relay information to mPFC, Amyg, and NAc.

Based on what we know about the mPFC, Amyg, and NAc and the features of ASD, we hypothesized that the effective functional connectivity among these regions in ASD patients is impaired. In order to test this hypothesis, we acquired resting-state functional magnetic resonance imaging (rs-fMRI) data from ASD patients and typically developing children, and used the Granger-causality-analysis (GCA) method to ascertain the efferent and afferent connections of these regions with the rest of the brain. The GCA method defines causal effects by examining whether the prior neural activity in one seed region predicts the activity in another region. GCA can be applied to time-series rs-fMRI data to identify the directional effective connectivity of functional interactions among brain regions (Guan et al., 2022), and we employed it as such.

Elucidating the effective functional connectivity of the socially pertinent mPFC, Amyg, and NAc in ASD will contribute to a comprehensive understanding of the neurobiological basis of social-preference abnormalities in ASD patients. Our experiments will investigate the mPFC, Amyg, and NAc together as a single unit and its association with a range of social behaviors, which could support the existence of a specific functional network responsible for guiding social preference in humans. Together, our results will hold significant importance for the future direction of therapeutic approaches for social rehabilitation in individuals with ASD.

## Materials and methods

### Study participants

The study included 37 children with ASD as well as a control group of 50 typically developing (TD) children, all of whom were obtained from the ABIDE database (ABIDE II, http://fcon_1000.projects.nitrc.org/indi/abide/) (Di Martino et al., 2017). The diagnosis of ASD patients was based on DSM-IV-TR criteria, which involved evaluating their developmental history, clinical observations, and current cognitive status. Additionally, their symptom severity was assessed using the autism diagnostic observation schedule-generic (ADOS-G) (Gotham et al., 2007) and the autism diagnostic interview-revised (ADI-R) tests (Lord et al., 2003). These criteria require that the child meet ADI-R cutoff for autism in the social domain and at least one other domain (communication and/or repetitive behaviors and restricted interests), and meet ADOS cutoff (autism or ASD) for the combined social and communication score. The intellectual level of all the children was evaluated using either the Wechsler Intelligence Scale for Children-Fourth Edition (WISC-IV) (Feis, 2003) or the Wechsler Abbreviated Scale of Intelligence (WASI), with intelligence quotient (IQ) readouts categorized under full-scale (FIQ), verbal (VIQ), and performance (PIQ) (Dumont et al., 2014). The ability of ASD patients to control responses during social interactions was also measured using social responsiveness scale (SRS) (Constantino et al., 2003). The SRS provides a total score and five subscales, which include Social Awareness (the ability to gather social information), Social Cognition (the ability to recognize and understand social information), Social Communication (the ability to respond to others’ speech), Social Motivation (the degree of motivation to engage in social behavior and interpersonal relationships), and Social Mannerisms (primarily involving stereotypy or repetitive behaviors). Higher SRS scores indicate greater severity of social impairment in ASD patients. The inclusion criteria for the children in the ASD group required that (1) they did not have any known severe medical, neurological, or psychiatric conditions aside from ASD, and (2) they had not taken any antipsychotic medications. The experimental protocol received approval from the local institutional review board, and written informed consent was obtained from the parents/guardians of each participant before the examination. Detailed scanning information, diagnostic procedures, and ethical statements are provided on http://fcon_1000.projects.nitrc.org/indi/abide/.

The exclusion criteria for study participation were the following: (1) Individuals from whom MRI scans were of poor quality, as identified through manual screening; (2) Individuals with excessive head movement, defined as a head displacement greater than 3 mm or a head rotation angle greater than 3°; (3) Individuals with a full scale intelligence quotient (FSIQ) below 80, as measured by either the WISC-IV or WASI; (4) Individuals with other neurological diagnoses such as epilepsy, as reported by their parents; (5) Individuals with contraindications for MRI, such as metallic implants or pregnancy.

### Data acquisition

Resting-state functional and structural MRI data were acquired at the Georgetown University Center for Functional and Molecular Imaging, utilizing a 3.0 Tesla scanner from Siemens Healthcare (Erlangen, Germany). Before scanning, participants underwent a simulator orientation to familiarize themselves with and adapt to the MRI scanning procedure. Participants were accompanied by their guardians throughout the entire scanning process, and supine participants used foam pads to stabilize their heads and minimize head movement. Functional imaging data were acquired using a standard echo-planar imaging (EPI) pulse sequence with the following scan parameters: repetition time (TR) = 2,000 ms, echo time (TE) = 30 ms, number of slices = 43, slice thickness = 2.5 mm, slice gap = 0.5 mm, flip angle = 90°, field of view (FOV) = 192 mm × 192 mm, matrix size = 64 × 64. T1-weighted three-dimensional images were acquired using a Magnetization Prepared Rapid Gradient Echo (MPRAGE) sequence with the following scan parameters: repetition time (TR) = 2,530 ms, echo time (TE) = 3.5 ms, number of slices = 176, slice thickness = 1 mm, slice gap = 0.5 mm, flip angle = 7°, field of view (FOV) = 256 mm × 256 mm, matrix size = 256 × 256.

### Data preprocessing

The functional imaging data were preprocessed using DPARSF 4.5 (Chao-Gan and Yu-Feng, 2010) from the DPABI V4.2 software package (http://rfmri.org/dpabi) (Yan et al., 2016) based on MATLAB R2014a (Math Works, Natick, MA, USA, www.mathworks.com). Several steps were performed to ensure the quality of the data and minimize artifacts:

(1) The initial ten timepoints of the functional images were discarded to achieve magnetic-field stability and allow participants to acclimate to the MRI scanner;
(2) An interval scan was utilized to avoid interference between adjacent layers, and the interpolation method was applied to approximate the actual result of each scan at a specific time point;
(3) Head-motion correction was conducted using functions in the SPM software package (http://www.fil.ion.ucl.ac.uk/spm) to address potential significant artifacts caused by excessive head motion in the fMRI time-series. The threshold for head motion was set at a mean framewise displacements (FD) -Jenkinson value of 0.2. This correction ensured that the brain was in the same position in each image, and also determined which individuals should be eliminated from further analysis (see exclusion criteria) (Huettel et al., 2008);
(4) To account for the large differences in brain size, shape, orientation, and gyral anatomy among participants, the corrected images were standardized to the Montreal Neurological Institute (MNI) standard space with a voxel size of 3 mm^3^ × 3 mm^3^ × 3 mm^3^. This standardization facilitated feasible comparisons between participants;
(5) Gaussian spatial smoothing (6 mm) was applied to all images to improve the signal-to-noise ratio (Duan et al., 2017; Wang et al., 2018);
(6) Linear-drift removal was performed using a linear model to eliminate systematic drift or trends in the signal that might arise from long-term physiological changes, residual motion-related noise after realignment, or instrument instability (Lowe and Russell, 1999);
(7) In order to minimize the impact of confounding variables, regression was performed on the white-matter signal, cerebrospinal-fluid signal, and whole-brain signal from each voxel;
(8) To reduce the influence of low-frequency and high-frequency components, a bandpass filter (0.01–0.08 Hz) was applied to all signals.

### Region of interest (ROI) extraction

**FIGURE 1:**
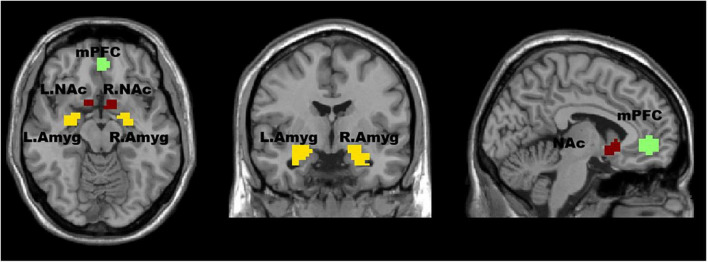
The regions of interest (ROIs) in this study are visualized in an axial (left image), coronal (middle image), and sagittal (right image) view of the brain. The medial prefrontal cortex (mPFC) is shown in red, the left (L) and right (R) nucleus accumbens (NAc) are shown in yellow, and the left and right amygdala (Amyg) are shown in orange. The ROI extraction in the mPFC was based on coordinates previously reported in the literature. The ROI extraction in the Amyg and NAc was based on the standard parcellation template.

The ROI extraction in the mPFC was based on coordinates previously reported in the literature, with a 10 mm sphere around the peak coordinates (–1, 47, –4) (Keshavan et al., 2014; Whitfield-Gabrieli et al., 2020). The ROI extraction in the Amyg and NAc was based on the standard parcellation template, specifically the Anatomical Automatic Labeling third edition (AAL3). Compared to the previous versions, AAL3 includes additional brain regions that had not been previously defined but are of interest in many neuroimaging studies. In the AAL3 template, the Amyg brain region is labeled as 45 (left) and 46 (right), while the NAc is a newly-added brain region located at 157 (left) and 158 (right). The REST toolbox (http://www.restfmri.net) was used to extract the Amyg and NAc regions. Figure 1 shows the ROIs chosen for mPFC, Amyg, and NAc.

### Fractional amplitude of low-frequency fluctuations (fALFF) analysis

The fALFF values of the ASD and TD groups of ROIs were calculated using DPABI software. Firstly, the blood oxygen level dependent (BOLD) signals from the two sets of ROIs were extracted. Next, a bandpass filter within the range of 0.01–0.1 Hz was applied to obtain the low-frequency oscillations. Following this, a Fourier transform was employed to convert the signals into the frequency domain. The power spectrum that has been obtained was run through the square root operation in order to derive the amplitude of low frequency fluctuations (ALFF). Subsequently, the mean amplitude spectrum within the range of 0.01–0.08 Hz was computed. This value was then normalized by dividing it by the mean ALFF value of the whole brain. The computed ALFF values were summed to obtain a total value. The fALFF value was obtained by dividing the sum of ALFF values by the sum of ALFF values within the entire frequency range. Finally, we normalized each voxel by dividing its fALFF value by the average fALFF value of the whole brain.

### Granger Causal Analysis

Granger Causality Analysis (GCA) is a multivariate, linear, autoregression-based method used to investigate the predictive relationship between time-series data. In our study, we selected the mPFC, bilateral Amyg, and bilateral NAc as ROIs and utilized the REST toolbox to compute the causal connections between these ROIs and the whole brain. The output of GCA is a coefficient matrix, where positive and negative coefficients represent excitatory or inhibitory effects between the regions, respectively. Specifically, positive coefficients indicate excitatory effects or positive feedback, while negative coefficients indicate inhibitory effects or negative feedback. Furthermore, the magnitude of these coefficients reflects the strength of the effect. To ensure that the coefficient matrix followed a normal distribution for statistical analysis purposes, a Fisher’s z transformation was applied. We analyzed the directed connectivity of resting-state networks between the three ROIs combined (x) and whole brain (y), taking into account the covariates of age, gender, and head-motion parameters (frame displacement; FD values).

### Statistical analysis

To perform statistical analysis on the demographic and behavioral data, SPSS 26.0 software was utilized. For continuous variables, a two-sample *t*-test with Bonferroni correction was performed, while for categorical variables, a χ^2^ test with Bonferroni correction was utilized, with a significance threshold set at *p* < 0.001. For the comparison of GCA effective connectivity between ROIs and the whole brain, as well as the fALFF values of the ASD and TD groups, a two-sample *t*-test was performed using SPM12 software. Multiple comparisons were corrected using cluster-level false discovery rate (FDR) correction. Moreover, Pearson correlation analysis was conducted to examine the relationship between GCA effective connectivity and SRS scores, with a significance threshold set at *p* < 0.05.

## Results

### Demographic and clinical information

Table 1 presents an overview of the demographic, intelligence, and behavioral measures for the two participant groups. The analysis showed no significant differences in demographics, including age (*t* = 2.70, *p* > 0.05), gender (χ^2^ = 1.94, *p* > 0.05), handedness (χ^2^ = 3.31, *p* > 0.05), and mean frame-displacement (*t* = 3.57, *p* > 0.05), as well as three categories of IQ levels (FIQ *t* = 0.57, *p* > 0.05, VIQ *t* = 0.00, *p* > 0.05, PIQ *t* = 0.02, *p* > 0.05). However, we observed that the ASD group exhibited significantly higher scores on the SRS, specifically in SRS_total (raw) (*t* = 151.68, *p* < 0.05), SRS_awareness (*t* = 88.92, *p* < 0.05), SRS_cognition (*t* = 193.44, *p* < 0.05), SRS_communication (*t* = 143.20, *p* < 0.05), SRS_motivation (*t* = 99.15, *p* < 0.05), and SRS_mannerisms (*t* = 164.31, *p* < 0.05), compared to the TD group (*p* < 0.001 for all subscales).

**TABLE 1:** Differences in demographic, intelligence, and behavioral measures between ASD and TD groups.

Data	ASD	TD	*t* or χ^2^ value	*p*-value
Age (years)	11.14 ± 1.45 (37)	10.57 ± 1.72 (50)	2.70^a^	0.104
Gender (M/F)	32/5	25/25	1.94^b^	0.163
Handedness (R/L)	30/7	47/3	3.31^b^	0.069
Mean FD_Jenkinson	0.1226 ± 0.0648	0.0994 ± 0.0498	3.57^a^	0.062
FIQ	118.50 ± 14.61 (36)	120.86 ± 14.12 (49)	0.57^a^	0.453
VIQ	121.11 ± 14.46 (36)	121.14 ± 15.53 (49)	0.00^a^	0.993
PIQ	115.32 ± 15.38 (25)	115.82 ± 13.42 (49)	0.02^a^	0.887
SRS_total (raw)	87.32 ± 35.25	19.42 ± 14.43	151.68^a^	**0.000** *
SRS_awareness	11.46 ± 3.81	4.76 ± 2.82	88.92^a^	**0.000** *
SRS_cognition	15.43 ± 5.69	2.80 ± 2.58	193.44^a^	**0.000** *
SRS_communication	28.89 ± 11.43	6.28 ± 5.98	143.20^a^	**0.000** *
SRS_motivation	13.78 ± 6.33	3.72 ± 2.88	99.15^a^	**0.000** *
SRS_mannerisms	15.97 ± 7.10	1.86 ± 2.78	164.31^a^	**0.000** *

Data are presented as the mean ± SD.

*p < 0.001 indicates significant differences between the groups.

^a^t-value was obtained by using the two-sample *t*-test.

^b^χ^2^ was obtained by using the chi-square test. Bold values indicate statistical significance. ASD, autism spectrum disorder; TD, typically developing; M, male; F, female; R, right; L, left; Mean FD_Jenkinson, mean frame displacement from Jenkinson algorithm; FIQ, full-scale IQ; VIQ, verbal IQ; PIQ, performance IQ; SRS, social responsiveness scale; SD, standard deviation.

### fALFF values of the ROIs

**FIGURE 2:**
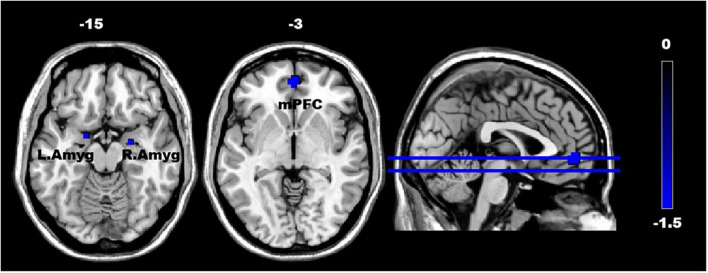
The difference in the fractional amplitude of low-frequency fluctuations (fALFF) in the regions of interest (ROIs) between the ASD and TD groups. The color scale on the right represents higher fALFF values in the cooler colors. Axial and sagittal views of the brain show decreased fALFF in the mPFC and bilateral amygdala. mPFC, medial prefrontal cortex; L. Amyg, left amygdala; and R. Amyg, right amygdala.

In the ASD group, the fALFF values demonstrated a decrease in the mPFC (*t* = 1.66, *p* < 0.05) (FDR-corrected) and bilateral Amyg (*t* = 1.66, *p* < 0.05) (uncorrected), as illustrated in Figure 2. Although the fALFF values in the NAc were slightly higher in the ASD group than in the TD group, no statistically significant differences were found.

### GCA of effective connectivity

**FIGURE 3:**
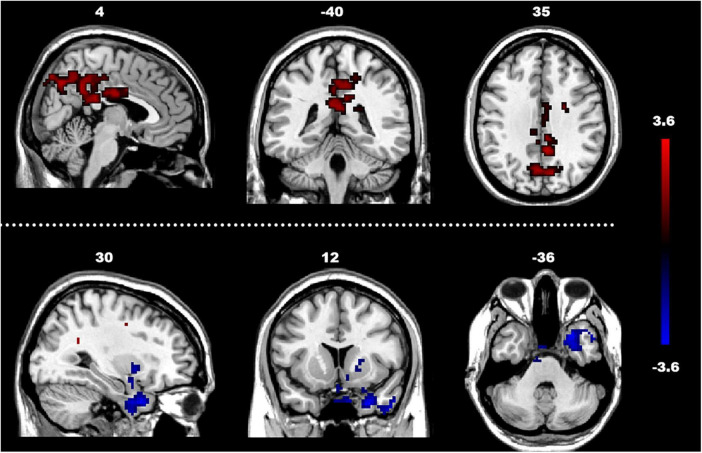
Between-group difference in GCA of connectivity from ROIs to whole brain (x to y) (FDR-corrected, cluster level *p* < 0.005). The color scale on the right represents higher GCA values in the warmer colors and lower ALFF values in the cooler colors. Sagittal (left image), coronal (middle image), and axial (right image) views of the brain show decreased GCA in the rSTG (blue) and increased GCA in rCG (red). R, right; L, left; P, posterior; A, anterior; ASD, autism spectrum disorder; and TD, typically developing; GCA, Granger Causality Analysis; rSTG, right superior temporal gyrus; rCG, right cingulate gyrus.

**FIGURE 4:**
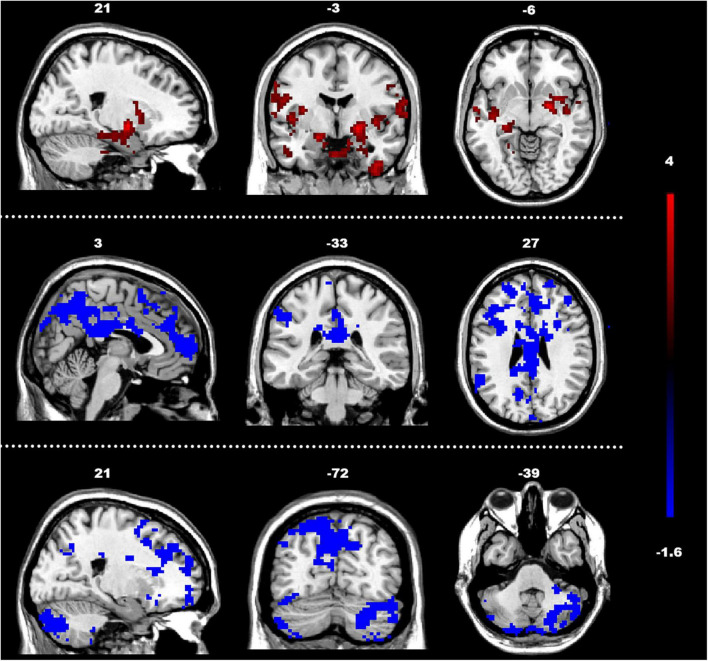
Between-group differences in GCA of connectivity from the whole brain to ROIs (y to x) (FDR-corrected, cluster level *p* < 0.005). The color scale on the right represents higher GCA values in the warmer colors and lower ALFF values in the cooler colors. Sagittal (left image), coronal (middle image), and axial (right image) views of the brain show decreased GCA in the rCbeCru1 and lCG (blue) and increased GCA in rGP (red). R, right; L, left; P, posterior; A, anterior; ASD, autism spectrum disorder; and TD, typically developing; GCA, Granger Causality Analysis; rGP, right globus pallidus; rCbeCru1, right cerebellar Crus1 area; lCG, left cingulate gyrus.

The analysis of the efferent connectivity from the ROIs to the whole brain revealed a significant increase in the right cingulate gyrus and a corresponding decrease in the right superior temporal gyrus when comparing the two experimental groups (*t* = 1.66, *p* < 0.05). More detailed results can be found in Table 2 and Figure 3. Conversely, in the analysis of the afferent connectivity to the ROIs, an increase was observed in the right globus pallidus while the right cerebellum Crus1 area and the left cingulate gyrus showed a decrease in ASD compared to the TD group (*t* = 1.66, *p* < 0.05). Detailed results can be found in Table 3 and Figure 4.

**TABLE 2:** Brain regions with significant differences in connectivity coming from the ROIs (ROIs efferent connectivity in ASD vs. TD).

Clusters	Voxels	Regions	MNI coordinates (x, y, z)	Cluster- level *p* (FDR-corrected)
1	759	rSTG	(30, 12, –36)	0.003
2	706	rCG	(4, –40, 35)	0.005

The MNI coordinates reported are the peak coordinates in the clusters of each seed. FDR, false discovery rate; rSTG, right superior temporal gyrus; rCG, right cingulate gyrus; ASD, autism spectrum disorder; TD, typically developing.

**TABLE 3:** Brain regions with significant differences in connectivity going to the ROIs (ROIs afferent connectivity in ASD vs. TD).

Clusters	Voxels	Regions	MNI coordinates (x, y, z)	Cluster- level *p* (FDR-corrected)
1	2,482	rGP	(21, –3, –6)	0.000
2	1,722	rCbeCru1	(21, –72, –39)	0.000
3	5,748	lCG	(3, –33, 27)	0.000

The MNI coordinates reported are the peak coordinates in the clusters of each seed. FDR, false discovery rate; rGP, right globus pallidus; rCbeCru1, right cerebellar Crus1 area; lCG, left cingulate gyrus; ASD, autism spectrum disorder; TD, typically developing.

### Correlation between GCA results and SRS scores

**FIGURE 5:**
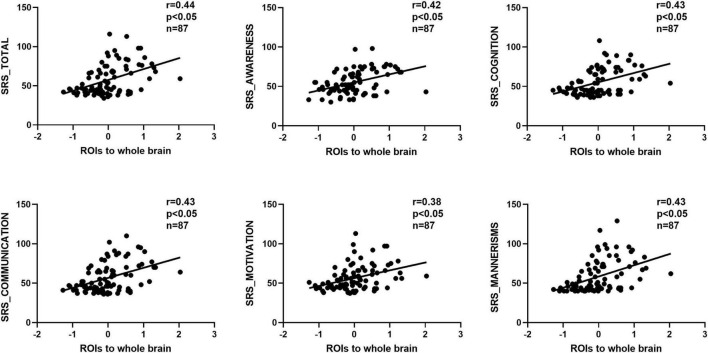
Correlation results between the effective connectivity from the ROIs to the whole brain (ROI efferents) and various domains of SRS scores. These correlations were found between the effective connectivity from the. Significant positive correlations were found with total scores (*r* = 0.44, *p* < 0.05), social awareness (*r* = 0.42, *p* < 0.05), social cognition (*r* = 0.43, *p* < 0.05), social communication (*r* = 0.43, *p* < 0.05), social motivation (*r* = 0.38, *p* < 0.05), and social mannerisms (*r* = 0.43, *p* < 0.05). SRS, social responsiveness scale.

**FIGURE 6:**
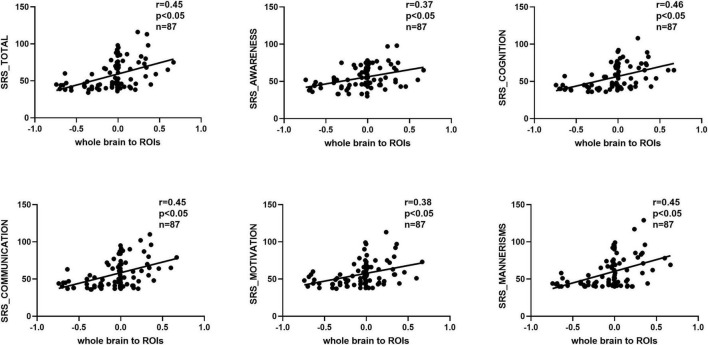
Correlation results between the effective connectivity from the whole brain to the ROIs (ROI afferents) and the aforementioned SRS domains. Significant positive correlations were found with total scores (*r* = 0.45, *p* < 0.05), social awareness (*r* = 0.37, *p* < 0.05), social cognition (*r* = 0.46, *p* < 0.05), social communication (*r* = 0.45, *p* < 0.05), social motivation (*r* = 0.38, *p* < 0.05), and social mannerisms (*r* = 0.45, *p* < 0.05). SRS, social responsiveness scale.

We observed significant positive correlations between GCA results and SRS scores (Figures 5, 6). Importantly, the GCA results were not correlated with other assessed indicators. These correlations were found between the effective connectivity from the ROIs to the whole brain (ROI efferents) and various domains of SRS scores (Figure 5). Specifically, significant positive correlations were found with total scores (*r* = 0.44, *p* < 0.05), social awareness (*r* = 0.42, *p* < 0.05), social cognition (*r* = 0.43, *p* < 0.05), social communication (*r* = 0.43, *p* < 0.05), social motivation (*r* = 0.38, *p* < 0.05), and social mannerisms (*r* = 0.43, *p* < 0.05). Additionally, there was a significant positive correlation between the effective connectivity from the whole brain to the ROIs (ROI afferents) and the aforementioned SRS domains (Figure 6). Specifically, we found significant positive correlations with total scores (*r* = 0.45, *p* < 0.05), social awareness (*r* = 0.37, *p* < 0.05), social cognition (*r* = 0.46, *p* < 0.05), social communication (*r* = 0.45, *p* < 0.05), social motivation (*r* = 0.38, *p* < 0.05), and social mannerisms (*r* = 0.45, *p* < 0.05).

## Discussion

In this study, we investigated the neural circuitry involving key brain regions (mPFC, Amyg, and NAc) in children with ASD, focusing on how these regions connect to the rest of the brain. We observed both increases and decreases in the efferent and afferent connectivity of these regions. We will discuss each result individually below. Our investigation also revealed a significant positive correlation between the effective connectivity among these regions in children with ASD and their SRS scores. These findings not only support our hypothesis that children with ASD present with impaired effective connectivity among these brain regions but also align with previous research (Chen et al., 2016). The aberrant patterns of effective connectivity that we revealed in our study could be attributed to potential anatomical abnormalities in these brain regions, which could serve as the foundation for autistic tendencies of social preference. Accordingly, our results provide novel perspectives on the neural basis for the atypical social-preference behaviors in children with ASD.

### Increased effective connectivity from the ROIs to the right cingulate gyrus in ASD

The cingulate gyrus, subdivided into anterior and posterior regions, is central to the brain’s processing of social information, decision-making, and emotional responses (Apps et al., 2013; Guo et al., 2019; Leung and Lau, 2020). Our findings enhance this understanding by revealing a nuanced pattern of neural activity in the cingulate gyrus of individuals with ASD. While the anterior cingulate cortex is linked to social behavior deficits in conditions such as in Shank3-mutant mice (Guo et al., 2019), our fALFF analysis identified weaker spontaneous BOLD signals in this region in ASD compared to TD groups. Despite this decreased activity, there was an increase in effective connectivity from the ROIs to the right cingulate gyrus, suggesting a compensatory mechanism, potentially because of enhanced feedback to the cingulate gyrus, contrasting with the typical hyper-connectivity observed in the posterior cingulate cortex in ASD (Lynch et al., 2013). Moreover, excessive activation was noted in the right cingulate gyrus during tasks such as online prosocial gaming (Chung et al., 2016), reflecting its role in connecting with medial cortical and temporal regions via the cingulum bundle (Hau et al., 2019). These adaptive changes could be influenced by genetic variations, such as those in the CNTNAP2 gene, which have been linked to abnormal cingulate activity and associated social awareness deficits in ASD (Chien et al., 2021).

### Decreased connectivity from the ROIs to the right superior temporal gyrus in ASD

The superior temporal gyrus plays a pivotal role in social functioning and has been extensively studied in ASD, where variations in neural activation have been noted across different contexts (Billeci et al., 2019; Peng et al., 2020; Su et al., 2021; Chen et al., 2022; Yue et al., 2022; Xiao et al., 2023). Previous research has consistently shown decreased activation in these regions during social-emotional communication and interaction tasks, correlating significantly with behavioral assessments in children with ASD (Su et al., 2021; Xiao et al., 2023). Moreover, regional homogeneity (ReHo) studies have observed reduced coherence in the left superior temporal gyrus, aligning with lower scores on the ADOS (Yue et al., 2022). Contrary to these findings, some fMRI studies report increased activation in the right superior temporal gyrus during social-animation tasks, suggesting a complex, task-dependent neural response (Chen et al., 2022). In our study, initial analyses using fALFF indicated a decrease in spontaneous neural activity within these regions in the ASD group compared with their TD peers. Further exploration using GCA revealed a significant reduction in effective connectivity from these ROIs to the right superior temporal gyrus, suggesting divergent neural adaptation in ASD. This finding underscores the decrease in typical activation patterns and enhances our understanding of the role of the superior temporal gyrus in ASD beyond the existing contradictory reports, indicating potential underlying mechanisms that may explain the variability observed in previous studies.

### Increased connectivity from the right globus pallidus to the ROIs in ASD

The globus pallidus, a crucial component of the mesencephalic dopaminergic pathway, plays a pivotal role in the brain’s reward circuitry and is intricately connected with the NAc. Although the fALFF values in the NAc did not show significant differences between the ASD and TD groups, we observed a noteworthy increasing trend in the ASD group. This aligns with previous observations of greater structural covariance within the right globus pallidus in children with ASD, which also noted a decrease in long-range structural covariance under conditions of enhanced local covariance (Duan et al., 2020). Expanding on these findings, our research uncovered a significant increase in effective connectivity from the right globus pallidus to the designated ROIs. This novel insight suggests an adaptive reorganization of neural connections that may compensate for the disrupted communication typically observed in the neural networks of individuals with ASD. By specifically linking these changes to the right globus pallidus, which is essential for reward processing and motivational aspects of behavior, our results contribute to a deeper understanding of how basal ganglia circuits may be uniquely reconfigured in ASD, potentially influencing the broader neurobiological underpinnings of the disorder.

### Decreased connectivity from the right cerebellar Crus I area to the ROIs in ASD

Recent research underscores the significant role of the cerebellum in regulating social behaviors, particularly within the context of neurological disorders such as ASD (Moreno-Rius, 2019; Van Overwalle et al., 2020). Voxel-based morphometry (VBM) studies have identified reductions in gray matter volume in the cerebellar Crus I/II in children with ASD, directly linking these anatomical changes to core symptoms of the disorder such as challenges in social interaction and communication (D’Mello et al., 2015). Additionally, observations of decreased functional connectivity in the right cerebellar Crus I, associated with language and socioemotional processing (King et al., 2019), have been noted in infants displaying delayed social communication skills (Okada et al., 2022). This pattern of connectivity provides new insights into potential mechanisms underlying difficulties with eye contact, an essential component of social interaction, corroborated by eye-tracking studies that relate these challenges to the volume of Crus I (Laidi et al., 2017). Moreover, our research highlights the dynamic interactions between the right cerebellar Crus I/II and the left prefrontal cortex, reinforcing their integral role in social cognition (Brady et al., 2020). Notably, our findings indicate that abnormalities in the right cerebellar Crus I may exacerbate core symptoms of ASD, suggesting that targeted structural and functional modifications within cerebello-cerebral circuits could be crucial in the disorder’s manifestation (D’Mello and Stoodley, 2015). Complementary animal studies have shown that stimulation of Purkinje cells in the right cerebellar Crus I can alleviate social preference deficits in PC-Tsc1 mutant mice, a model for ASD (Kelly et al., 2020). These results not only confirm but also expand upon existing literature by illustrating how specific interventions in these circuits can modulate social behaviors.

### Decreased connectivity from the left cingulate gyrus to the ROIs in ASD

Previous research has demonstrated abnormal functional connectivity in the left posterior cingulate cortex of ASD patients, often characterized by decreased long-range or interhemispheric connections coupled with increased short-range or intrahemispheric connections (Venkataraman et al., 2015). Similarly, studies of brain white matter structural networks have shown that individuals with ASD exhibit alterations in node efficiency within the left cingulate gyrus, linked to the severity of social impairment symptoms (Qian et al., 2018). Building upon these findings, our study observed a novel pattern: although there was weakened spontaneous neural activity in the ROIs, we detected a significant increase in effective connectivity from these ROIs to the right cingulate gyrus. This increase contrasts with a decline in effective connectivity from the left cingulate gyrus to the ROIs, suggesting a sophisticated mechanism of targeted regulation through negative feedback from the left cingulate gyrus. This mechanism points to the presence of a compensatory feedback loop, effectively activated under the impaired connectivity conditions typical in ASD. This discovery not only corroborates previous findings but also advances our understanding by illustrating how these compensatory mechanisms may function distinctly within different regions of the cingulate gyrus in response to ASD-specific neural challenges. Such insights provide a clearer picture of the complex neural dynamics involved in ASD and underscore the nuanced roles of various cingulate regions in modulating social behaviors.

### Effective connectivity of the ROIs correlates with SRS scores in ASD

The bi-directional abnormalities in effective connectivity that we revealed, both ROIs’ afferents and efferents, showed a positive correlation with SRS scores. This important result implies that impaired connections within the mPFC, Amyg, and NAc contribute to social preference deficits in ASD children. It also serves to confirm our choice of ROIs as brain regions crucial for social behavior. Taken together with the known involvement of these three brain regions with social cognition, emotional responses, social reward, and social decision-making (Kelly et al., 2020; Fortier et al., 2022), our study supports the existence of a functionally interconnected network specifically responsible for social preference behavior.

Every scientific study has limitations that must be acknowledged in order to appropriately understand its results. In our study, the relatively small sample size calls for future research with larger cohorts to improve the reliability and reproducibility of the data. Additionally, ASD is a heterogeneous disorder with varying symptomatic profiles that we did not take into account in our experimental design, as our ASD group was not subdivided by levels of autistic severity. It is possible that different neural mechanisms may underlie the different subtypes or functionality types in ASD. Finally, our analysis focused on the selected ROIs (mPFC, Amyg, and NAc) as a single entity because we were interested in examining the connectivity as a whole with the rest of the brain; we did not examine the connectivity of each individual seed point to the whole brain. Although we computed the differences in fALFF values between the two groups when analyzing the effective connections, the results of bilateral amygdala were uncorrected. These limitations will be addressed in future research. Future research should also investigate the structural connections corresponding to the functional connectivity changes as this additional knowledge would greatly contribute to a comprehensive understanding of the neural basis of ASD.

## Conclusion

The results of our study demonstrate that the effective connectivity of the mPFC, Amyg, and NAc is impaired in ASD brains, and that these impairments correlate with decreased social responsiveness behaviors in autistic children (i.e., increased severity in autistic tendencies). Ultimately, these findings provide valuable insights into the underlying neural pathophysiology of social preference in children with ASD and can inform new approaches to their rehabilitation.

## Data availability statement

Publicly available datasets were analyzed in this study. This data can be found here: http://fcon_1000.projects.nitrc.org/indi/abide/.

## Ethics statement

The studies involving humans were approved by the Institutional Review Boards (IRBs) of Children’s National Medical Center and Georgetown University, and the local ethics committee of Dongguan Eighth People’s Hospital (Dongguan Children’s Hospital) under reference AF/SC-15/01.0. The studies were conducted in accordance with the local legislation and institutional requirements. Written informed consent for participation in this study was provided by the participants’ legal guardians/next of kin.

## Author contributions

SD: Funding acquisition, Investigation, Methodology, Writing – original draft. ST: Methodology, Software, Writing – original draft. CG: Data curation, Investigation, Writing – review & editing. YL: Methodology, Writing – review & editing. XL: Conceptualization, Funding acquisition, Supervision, Writing – review & editing.
